# Influence of cervicovaginal microbiota on *Chlamydia trachomatis* infection dynamics

**DOI:** 10.15698/mic2025.04.848

**Published:** 2025-04-15

**Authors:** Emily Hand, Indriati Hood-Pishchany, Toni Darville, Catherine M. O'Connell

**Affiliations:** 1 Department of Microbiology and Immunology, University of North Carolina at Chapel Hill.; 2 Department of Pediatrics, University of North Carolina at Chapel Hill.

**Keywords:** chlamydia, gonorrhea, lactobacilli, microbiome, 16S rRNA, immunity, multiomics

## Abstract

The cervicovaginal microbiome (CVM) is increasingly being considered as an important aspect of women’s health, particularly in relation to the risk and progression of sexually transmitted infections (STIs). CVM composition varies significantly between individuals and is shaped by factors including diet, age, environmental exposures, and lifestyle. Understanding these influences may shed light on how microbial imbalances contribute to infection susceptibility and the development of reproductive health disorders. Five distinct community state types (CSTs) classify common CVM compositions. Most CSTs (I, II, III, V) are characterized by a dominant *Lactobacillus* species and are associated with better or neutral reproductive health, including reduced coincident detection of STIs such as *Chlamydia trachomatis*. In contrast, CST IV is composed of diverse, predominantly anaerobic, microbial species and is associated with CVM dysbiosis, bacterial vaginosis, and a heightened risk of STI acquisition. This review examines the complex interplay between the CVM, *C. trachomatis* infection, and host immune responses, highlighting the role of metabolites such as short-chain and long-chain fatty acids, indole, and iron in modulating pathogen survival and host defenses. Additionally, the impacts of CVM composition on *C. trachomatis* persistence, ascension, and clearance are discussed, alongside co-infection dynamics with pathogens like *Neisseria gonorrhoeae* and *Mycoplasma genitalium*.

## Abbreviations

CPAF - Chlamydial Protease Activity Factor,

CSTs - community state types,

CVM - cervicovaginal microbiome,

EBs - elementary bodies,

LCFAs - long chain fatty acids,

PID - pelvic inflammatory disease,

PMNs - polymorphonuclear leukocytes,

RBs - reticulate bodies,

SCFAs - short chain fatty acids,

STIs - sexually transmitted infections,

TLR - Toll-like receptor,

TRAC - T cell Response Against Chlamydia,

VMT - vaginal microbiota transplant.

## INTRODUCTION

A microbiome is a collective community of microbes—including bacteria, viruses, archaea, and fungi—that inhabit a specific host environment. Over the past two decades, research has increasingly focused on the gut microbiome, yielding significant discoveries regarding the impact of its microbial communities on human health and disease. Advances in this field have enabled translational applications, such as fecal transplants and probiotic therapies, which have proven effective in managing conditions like *Clostridium difficile *infection, ulcerative colitis, and recurrent urinary tract infections 1,2,3,4. The gut microbiome is a highly diverse ecosystem, with its composition varying across different populations and individuals. However, healthy gut microbiomes are typically dominated by members of the *Firmicutes *and *Bacteroidetes *phyla, along with* Actinobacteria* and *Proteobacteria* 5,6,7. In a study analyzing ~3400 healthy adults, the relative abundance of *Firmicutes* ranged widely from 6% to 100%, while *Bacteroidetes *varied from 0% to 90%, underscoring the diverse gut microbiome compositions present within healthy populations 8. Numerous factors—such as diet, environment, and physical activity—shape this variability in gut microbiome composition, contributing to the individual diversity seen across populations. Additionally, the gut microbiome composition has been linked to host immune functions, influencing disease progression and resolution 9. Gut microbes can inhibit opportunistic pathogens and regulate innate and adaptive immune cell functions. Understanding these interactions may offer insights into the dynamics of other less intensively studied human microbiomes, such as the cervicovaginal microbiome (CVM).

**Figure 1:**
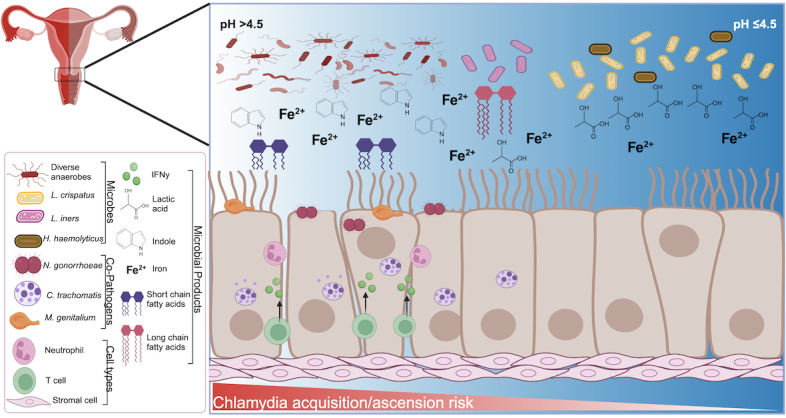
FIGURE 1: Summary of cervicovaginal microbiome risk factors associated with *Chlamydia trachomatis* infection. A summary of microbes, microbial products, and co-pathogens in the CVM that are associated with increasing risk of C. trachomatis infection acquisition and ascension.

The CVM is a dynamic microbial community with lower overall diversity than the gut microbiome 5. Factors such as menstruation, age, contraceptive use, hormones, and sexual activity shape CVM composition 10,11. The CVM plays a pivotal role in reproductive health and disease, influencing susceptibility to sexually transmitted infections (STIs) such as *Chlamydia trachomatis* (**Figure 1**). The composition of the CVM is classified into community state types (CSTs), based on the dominant bacterial species present. In a study using 16s rRNA gene sequencing, Ravel *et al*. identified five common CSTs (I-V) in a cohort of 396 women 12. CSTs I, II, III, and V are each dominated by a single *Lactobacillus* species (*L. crispatus, L. gasseri, L. iners, and L. jensenii*, respectively) 12 and are typically associated with low microbial diversity and positive reproductive health outcomes 13,14,15. In contrast, CST IV, characterized by diverse anaerobic bacterial genera with low *Lactobacillus *presence 12, is linked to adverse conditions such as bacterial vaginosis 16, pregnancy loss 17, and pre-term birth 18. New classifications and sub-classifications within CSTs are emerging with advancements in methodology and larger, more diverse sampling efforts 19,20,21.

This review examines the complex interplay between the CVM, *C. trachomatis *infection, and host immune responses, highlighting the role of metabolites such as short-chain and long-chain fatty acids, indole, and iron in modulating pathogen survival and host defenses. Additionally, the impacts of CVM composition on chlamydial persistence, ascension, and clearance are discussed, alongside co-infection dynamics with pathogens like *Neisseria gonorrhoeae* and *Mycoplasma genitalium*.

*Lactobacillus *species are present in both the gut and CVM, and it has been proposed that strains resident in the CVM originated in the gut 22. Analyzing site-specific data is important because of the unique roles that the gut microbiome and the CVM play in host health. At both mucosal sites, lactic acid-producing *Lactobacilli* contribute to maintaining homeostasis and reducing inflammation 23,24,25,26. Short chain fatty acids (SCFAs) are metabolic by-products including acetate, propionate, butyrate, and succinate, which are produced by microbes present in both the gut microbiome and the CVM 27. However, the abundance and function of SCFAs differ between these sites. In a healthy gut, SCFAs such as butyrate and propionate—primarily produced by *Firmicutes* and *Bacteroides* -support a low pH, promoting growth of beneficial *Lactobacillus *species that protect against pathogen colonization 28. SCFAs have anti-inflammatory properties, enhancing antimicrobial peptide production and mucus secretion, reducing the risk of bacterial translocation, or “leaky gut” 5,29,30. Conversely, CVM-derived SCFAs are linked to dysbiosis and tend to trigger pro-inflammatory responses, often associated with bacterial vaginosis 31,32,33. These contrasting effects may be due to variations in SCFA concentrations, microbial interactions, or the cell types present in each environment. Thus, while similar SCFAs are generated within both microbiomes, their physiological impacts diverge significantly depending on the site.

Like the gut microbiome, the CVM modulates immune responses, influencing the host’s capacity to prevent or resolve infections. Research has shed light on the impact of microbiome composition on the establishment, persistence, and clearance of STIs, affecting susceptibility to pathogens like human immunodeficiency virus (HIV) 34, herpes simplex virus 35, *Mycoplasma genitalium, *and *C. trachomatis *infection 14,36. CVM composition has been shown to affect the risk of acquiring *C. trachomatis,* with active chlamydial infection in turn altering CVM dynamics 14,36,37,38. *C. trachomatis* infection activates the host’s innate and adaptive immune responses, notably increasing neutrophil presence and upregulating T cell cytokine production (reviewed in 39,40). Technologic advances and multiomic approaches are expanding opportunities to study the complex interactions of host, pathogens, and CVM, enhancing our understanding of how these interactions influence health outcomes.

## *C. TRACHOMATIS* DEVELOPMENT

*C. trachomatis* is an obligate intracellular pathogen and the most prevalent bacterial STI worldwide 41. Approximately 1.6 million cases were reported in 2022 in the United States, 42, though this figure was likely underestimated due to limited screening during the Covid-19 pandemic 43. Although antibiotic treatments are effective at clearing *C. trachomatis *infections, 70% of cases in individuals with a uterus are asymptomatic, and often remain undiagnosed 44. If untreated, infection can ascend from the cervix to the upper genital tract, leading to complications such as pelvic inflammatory disease (PID), chronic pelvic pain, ectopic pregnancy, and tubal factor infertility 41. In individuals with a penis, around 50% of infections are asymptomatic, and untreated cases can result in urethritis, proctitis, and epididymitis 45,46. There are multiple serovars of *C. trachomatis* comprising two major biovars. Strains of serovar A, B, and C cause ocular trachoma, while serovars D through K cause genital infections 47. Strains belonging to serovars L1, L2, and L3 cause lymphogranuloma venereum 47.

*C. trachomatis *has a biphasic developmental cycle that begins when infectious extracellular elementary bodies (EBs) attach to host epithelial cells 48. EBs adhere to the host cell and induce invagination of the host cell membrane, forming an intracellular protective vacuole called an “inclusion.” Chlamydial effector proteins remodel and expand the inclusion and EBs differentiate into metabolically active reticulate bodies (RBs), which depend on host cell metabolites for essential nutrients and energy intermediates to sustain replication 49. After replication, RBs redifferentiate into infectious EBs, which exit the host cell either by cell lysis or extrusion, to continue the infection cycle in adjacent cells or a new host 50. Alternatively, RBs may enter a “persistent” state, forming aberrant bodies under conditions of nutrient deprivation or antibiotic exposure 51,52,53,54. Aberrant RBs exhibit reduced metabolic activity, ceasing division but remaining viable. When conditions improve, aberrant forms revert to normal development, producing infectious progeny to sustain the infection 55,56,57.

The host immune response targets *C. trachomatis *replication by exploiting the pathogen's dependence on tryptophan 52. A critical mechanism of chlamydial inhibition and immune defense is the production of IFNγ by natural killer cells and adaptive T cells 51,58,59,60. IFNγ reduces tryptophan availability via the indoleamine 2,3-dioxygenase (IDO) enzyme 51,61, thus encouraging the formation of chlamydial aberrant bodies 51,52. Iron is another critical nutrient for *C. trachomatis *survival and its depletion similarly triggers aberrant body formation 62,63. Additionally, chlamydia-specific T cells produce tumor necrosis factor-α (TNFα), which further inhibits chlamydial infection by activating oxidative pathways in epithelial cells and phagocytes, and by stimulating adaptive T cell responses 64. Together, these immune responses limit the pathogen's replication and promote immune protection.

## MECHANISMS OF CERVICOVAGINAL MICROBIOTA MODULATION OF *C. TRACHOMATIS* INFECTION

### *Lactobacillus* species 

*Lactobacillus *species predominate four of the five commonly described CSTs. *Lactobacillus-*dominated CVMs are typically considered “healthy,” and are often protective against STIs, including *C. trachomatis *14,15,65. The protective effects of *Lactobacillus *are thought to stem from several mechanisms. First, they act directly against *C. trachomatis *through production of lactic acid 66, which reduces the cervicovaginal pH, creating an inhospitable environment. Additionally, a robust *Lactobacillus* community may outcompete *C. trachomatis *and other pathogens for essential nutrients, limiting their ability to establish infection 27. Finally*,* *Lactobacillus* species contribute to CVM homeostasis by promoting an anti-inflammatory environment 27,67. The presence of *L. crispatus* and *L. jensenii, *for instance, is associated with decreased expression of pro-inflammatory cytokines such as IL-6, IL-8, and TNFα 24,27,68. These combined mechanisms support a healthy CVM and help reduce susceptibility to STIs.

Not all *Lactobacillus *species offer the same level of protection against *C. trachomatis*. Among *Lactobacillus *species*, L. crispatus *(CST I) demonstrates the strongest protective effect against *C. trachomatis *65,69,70. However, CST III, which is dominated by *L. iners*, has been linked to a higher incidence of *C. trachomatis *infections 13,14,38,69. Unlike other CVM-associated *Lactobacillus* species that produce D-and L-lactic acid, *L. iners* synthesizes only L-lactic acid 71. D-lactic acid has stronger antimicrobial properties, more effectively inhibiting pathogens than L-lactic acid 14,72,73. *In vitro*, D-lactic acid also inhibits epithelial cell proliferation, which normally promotes chlamydial infection 14. In *L. iners*-dominated CVMs, a low D- to L-lactic acid ratio correlated with increased epithelial production of the extracellular matrix metalloprotease inducer (EMMPRIN) 72, that upregulates matrix metalloprotease-8 (MMP-8). This activity undermines extracellular matrix integrity, facilitating pathogen spread to the upper genital tract 72. *L. iners* does not produce hydrogen peroxide (H_2_O_2_)_, _a by-product of many other *Lactobacillus *species that may also contribute to protection against STIs and bacterial vaginosis-associated microbes 74. However, *in vitro* studies suggest that lactic acid, rather than H_2_O_2_, is the primary anti-chlamydial metabolite produced by *Lactobacillus *66,75. Thus, *L. iners*' lack of H₂O₂ production does not fully explain its comparatively weaker protective effects against *C. trachomatis*.

### Fatty acids

Reduced lactic acid levels, CST IV composition, and bacterial vaginosis have all been linked to higher rates of *C. trachomatis *infection 15,59,76. Some of the anaerobic microbes commonly associated with CST IV produce SCFAs 5,77, which may negatively affect the cervicovaginal cellular environment 77. Supporting evidence from human cohort data indicates that increased SCFA production correlates with CVM dysbiosis and reduced lactic acid abundance 33,77,78. Since SCFAs are mainly by-products of CST IV-associated microbes, their prevalence may outcompete lactic acid-producing *Lactobacillus*, leading to a shift in overall lactic acid levels that dampens the environmental defense against STIs.

SCFAs produced by bacterial vaginosis-associated microbes in the CVM may alter the host immune response to influence susceptibility to *C. trachomatis *and may increase or decrease infection risk, depending on whether the SCFAs exert pro- or anti- inflammatory effects. Generally, CVM-derived SCFAs are considered pro-inflammatory, which tends to increase susceptibility to STIs. However, data on SCFA-related immune modulation in the lower female genital tract has been contradictory. For instance, when a monocytic cell line was treated with different SCFAs, succinic acid strongly inhibited chemotaxis, while acetic acid only slightly reduced it 25. Inhibiting chemotaxis reduces immune cell recruitment to infection sites, dampening inflammation and limiting the immune response.

SCFAs in the gut microbiome are well-known for their anti-inflammatory effects. They play a crucial role in maintaining intestinal health by regulating immune responses, preserving the intestinal barrier, and promoting the development and function of regulatory T cells. 29,79,80. In a healthy gut, SCFAs—primarily acetate, propionate, and butyrate—are produced at relatively high concentrations, ranging from 20-140 mM, depending on the specific SCFA and location within the intestine 81. These high levels contribute to a balanced immune environment, protection against pathogens, and enhanced epithelial barrier function.

In contrast, CVM-derived SCFAs appear to have different physiological consequences, likely due to significant concentration differences and the unique environment of the vaginal mucosa. Under normal conditions, vaginal SCFA levels are very low (0-10 mM). However, during dysbiosis, such as bacterial vaginosis, these levels can increase to 20 mM or higher 25,82. Unlike in the gut, elevated SCFA concentrations in the cervicovaginal region are often associated with inflammation, disruption of the vaginal epithelial barrier, and increased susceptibility to infections. These effects may stem from the acidic pH and distinct immune landscape of the vagina, which differ markedly from the gut environment. The differing concentrations and local environments suggest that SCFAs may shift from anti-inflammatory roles in the gut to potentially pro-inflammatory or disruptive roles in the CVM. However, the precise mechanisms underlying these contrasting effects remain poorly defined and are an important area for future research. Understanding how SCFAs influence immunity and barrier function in the CVM compared to the gut could provide key insights into microbial dysbiosis and related health outcomes.

An *in vitro* study by Mirmonsef, *et al*. illustrates how SCFA concentrations impact immune function 83. When peripheral blood mononuclear cells and neutrophils were exposed to high concentrations (20 mM) of acetic and butyric acids, they showed increased pro-inflammatory cytokine production (IL-8, IL-6, and IL-1b) 83. However, at low concentrations (0.02-2 mM), these SCFAs did not trigger inflammatory cytokine production 83. Moreover, low levels of SCFAs combined with Toll-like receptor (TLR) ligands prompted pro-inflammatory responses, specifically IL-8 and TNFα, suggesting that SCFAs might interact with the immune environment to enhance inflammation 83. Interestingly, similar tests using neutrophils showed that TLR ligands with SCFAs mildly induced IL-8 and had no effect on TNFα production 83, highlighting that cell type, SCFA concentration, and SCFA type all affect the immune response. These findings suggest that SCFAs are not solely responsible for the pro-inflammatory environment associated with CST IV and bacterial vaginosis, which are both linked to a heightened risk of *C. trachomatis *infection. Instead, it’s likely that other CVM metabolites contribute to this environment.

Delgado-Diaz, *et al*., investigated the effect of SCFAs on pro-inflammatory cytokine responses *in vitro* using three physiologically relevant cell types - ectocervical epithelial (Ect), vaginal epithelial (VK2), and primary epithelial cells recovered from cervicovaginal tissue - by administration of acetic acid, butyric acid, or succinic acid and/or TLR stimulation 67. They determined that mixed SCFA concentrations (acetic acid 100 mM, butyric acid 4 mM, succinic acid 20 mM, propionic acid 4 mM) increased TNFα production in all three cell types, but only after prolonged exposure (24 hr). The researchers further observed cell-type-specific responses when mixed SCFAs were combined with TLR agonists. Although SCFAs potentiated TLR-induced TNFα production in all three cell types, only Ect cells produced IL-8, while secretion of RANTES and IP-10 was inhibited across all cell types. Administration of acetic acid alone induced TNFα production by Ect and VK2 cells but not primary cells. These findings suggest that immune modulation in response to SCFA is multifactorial, influenced by cell type, SCFA in combination versus alone, concentration, pH, exposure duration, and simultaneous presence of TLR agonists (e.g., viral or bacterial pathogens). This study highlights the importance of using physiologically relevant models to understand how SCFAs and CVM composition influence immune response and STI susceptibility.

Elevated levels of long chain fatty acids (LCFAs) in cervical fluid - including pentadecanoic, heptadecanoic, and oleic acids - have been associated with *C. trachomatis *infection 77. In a metabolomic study by Borgogna, *et al*., women with *C. trachomatis *or *C. trachomatis/M. genitalium* coinfection exhibited significantly higher LCFA concentrations compared to uninfected participants 77. Microbes that produce LCFAs, particularly anaerobes such as *Prevotella *species, are abundant in CST IV 25. CT’s dependence on LCFAs for maintaining its inclusion and sustaining infection has been well documented 84. Extracellular LCFAs are transported into the inclusion via acyl carrier proteins and are available for use by replicating chlamydiae 85. The correlation between increased LCFA levels in cervical fluid during *C. trachomatis *infection and the pathogen’s reliance on these fatty acids suggests that a CVM rich in LCFA-producing anaerobes could support *C. trachomatis *infection. Despite the theorized benefit that a LCFA-enriched CVM might provide to *C. trachomatis*, recent findings by Zhu, *et al*. suggest a protective, anti-bacterial vaginosis role for LCFAs, specifically oleic acid 86. Since bacterial vaginosis is an established risk factor for *C. trachomatis *infection, their findings imply that oleic acid supports a CVM that is less conducive to *C. trachomatis *proliferation. In their *in vitro* study, Zhu, *et al*. demonstrated that supraphysiological levels of oleic acid inhibited growth of bacterial vaginosis-associated (CST IV) anaerobes and *L. iners* (CST III), while enhancing *L. crispatus *(CST I), the species considered to be most protective against *C. trachomatis *infection 86.

Overall, current data does not provide a definitive conclusion regarding the role of LCFAs on susceptibility to *C. trachomatis *infection and subsequent clearance. Although it seems increased levels of LCFAs tend to be conducive to *C. trachomatis *infection, it is not clear how the addition of supraphysiological levels of LCFAs may impact *C. trachomatis *acquisition and/or ascension. Fatty acids perform multiple functions, and their effects may vary depending on anatomical location, cellular interactions, and external influences (e.g., infection status, diet). Additionally, the contribution of fatty acids from the host itself are not clearly defined, thus fatty acids in the CVM could be both host and microbe derived. These mixed findings highlight the need for additional *in vitro* and *in vivo* studies to clarify the causal and mechanistic impacts of LCFA and SCFA concentrations on *C. trachomatis *during natural infection. Broadening focus to the metabolome could provide more nuanced insights into the association between metabolomic by-products, CVM composition, and infection risk.

### Indole

*C. trachomatis* relies on host-derived tryptophan for replication. Natural killer cells, macrophages and chlamydia-specific T cells release IFNγ, which induces production of indoleamine 2,3-dioxygenase (IDO), an enzyme that degrades tryptophan, limiting its availability for *C. trachomatis. *Since *C. trachomatis *cannot synthesize tryptophan *de novo*, intracellular depletion leads to growth arrest. Under extreme tryptophan starvation and IFNγ exposure, *C. trachomatis* enters a persistent state characterized by viable but non-replicating forms 87,88. However, genital *C. trachomatis *serovars can adapt by expressing a functional tryptophan synthase, encoded by *trpBA *89,90, that can utilize indole, a CVM-generated intermediate 91,92,93. CST IV is populated with diverse indole-producing anaerobes, including *Prevotella spp. *12. This adaptation has enabled *C. trachomatis *to better evade IFNγ-induced tryptophan depletion in an environment rich in *Prevotella intermedia *and *Prevotella nigrescens* 58,59. Furthermore, anaerobes, including those that produce indole, raise the CVM pH 51, creating an environment more conducive to colonization by a variety of pathogens, including *C. trachomatis*.

### Iron 

*C. trachomatis *depends on its host cell to obtain essential nutrients, including iron 62. Iron plays a crucial role in survival for nearly all organisms, as it supports biochemical processes such as the electron transport chain and tricarboxylic acid cycle. In host-pathogen interactions, the host may employ “nutritional immunity” to limit iron availability, restricting pathogens’ access to this essential resource as a defense mechanism 94. Under low-iron conditions, *C. trachomatis* adopts a persistent state to survive; transcriptional studies have shown that *C. trachomatis *in iron-deprived environments redirects its transcriptional focus from replication to nutrient storage, a strategy that helps it endure until environmental conditions improve 95.

Iron scarcity also impacts host cell metabolism and gene expression, indirectly affecting the supply of other substrates required for chlamydial development 96. While iron is vital for *C. trachomatis *development, the mechanisms employed by *C. trachomatis *to respond to iron scarcity and iron acquisition are not entirely understood. *C. trachomatis *lacks many typical bacterial pathways for iron uptake, complicating our understanding of its iron-acquisition strategies. However, proteins encoded by the genes of the *ytgABCD* operon are thought to play a central role in *C. trachomatis *iron acquisition (extensively reviewed by 62), and *C. trachomatis *exploits the host-derived iron-binding protein transferrin to deliver iron to its intracellular inclusion using secreted effectors 62.

Activation of *trpBA* transcription enables *C. trachomatis *to scavenge environmental indole and synthesize tryptophan, helping *C. trachomatis *evade the host's IFNγ response 85. A 2019 study by Pokorzynski *et al*. showed that *trpBA* expression also depends on iron via the YtgR regulator, suggesting a critical link between iron and tryptophan availability 97. When iron levels are low, YtgR cannot bind to the *trpRBA* intergenic region, allowing *trpBA *transcription and indole salvage. When iron is sufficient, YtgR represses *trpBA* transcription, thus halting tryptophan synthesis 97. YtgR also responds to tryptophan levels, creating a regulatory feedback loop that modulates *trpBA* expression in response to both iron and tryptophan availability. A unique WWW codon motif present in the proximal region of the promoter for coordinately regulated *C. trachomatis *genes detects tryptophan scarcity, fine-tuning YtgR activity even when indole is unavailable 98. This regulatory system is advantageous in the CVM environment, which is typically indole-rich but iron-poor. Although *C. trachomatis*’ iron-acquisition mechanisms remain unclear, YtgR is associated with the *ytgABCD* operon, specifically YtgCD permease 93, which likely plays a role. Further studies into *C. trachomatis*’ direct iron-acquisition mechanisms and genetic regulation could deepen our understanding of its pathogenesis.

*C. trachomatis *infection often co-occurs within a CVM rich in anaerobic, indole-producing microbes and characterized by low iron levels 97. In this environment, iron scarcity serves as a signal for *C. trachomatis *to upregulate tryptophan synthase, enabling efficient metabolism of indole—circumventing the host's IFNγ-driven immune response. This interplay between iron and tryptophan depletion reveals interconnected stress responses that appear to support *C. trachomatis *survival during host immune pressures.

To understand *C. trachomatis *survival, it is essential to consider both how *C. trachomatis *adapts to host-induced stress and how the CVM may impact either host or *C. trachomatis *responses. *Lactobacillus* species, often prevalent in a healthy CVM, have minimal iron requirements relative to other bacteria 99,100,101. This suggests that *Lactobacillus* species persist regardless of fluctuations in iron availability within the CVM. In contrast, anaerobes associated with bacterial vaginosis and *C. trachomatis *generally have higher iron requirements for growth. Host physiology influences iron availability in the lower genital tract, with iron levels naturally fluctuating throughout the menstrual cycle. These changes could impact *C. trachomatis *infection success at different cycle stages. An additional host-derived variable is anemia. Interestingly, dysbiotic CVMs—characterized by an overgrowth of bacterial vaginosis-associated bacteria--have been linked to iron deficiency in pregnant women 102. This association may seem counterintuitive, given that many bacterial vaginosis-associated bacteria have high iron requirements. However, it is possible that these bacteria have evolved efficient iron-scavenging mechanisms that allow them to thrive even in iron-limited conditions, such as through siderophore production or the utilization of host-derived heme sources. Additionally, iron limitation may alter host immune responses or vaginal epithelial integrity in ways that favor dysbiosis. This paradox underscores the need for further human cohort studies to investigate the interplay between CVM composition, *C. trachomatis* incidence, and the specific metabolite landscape of both the CVM and the host. A deeper understanding of these dynamics could provide insights into how microbial communities persist and influence infection risk under varying iron availability.

In our recent study investigating CVM composition and chlamydial ascension risk, we identified *Haemophilus haemolyticus* as one of eleven CVM members that, in combination, were predictive of reduced likelihood of *C. trachomatis *reaching the endometrium 103. In the context of the human respiratory tract, *H. haemolyticus* is a protective commensal that outcompetes pathogenic non-typeable *H. influenzae*, because of its superior ability to sequester iron 104. If *H. haemolyticus* acts similarly in the context of CVM, it may constrain chlamydial replication, ultimately limiting ascension. Future studies on CVM should incorporate iron profiling to further elucidate this connection.

A study in Burkina Faso examined the impact of oral iron supplementation on lower genital tract infections and CVM composition 105. The researchers hypothesized that increased iron availability might heighten the risk of genital tract infections or dysbiosis. However, findings indicated that iron supplementation did not significantly elevate infection or dysbiosis risk. Notably, iron biomarkers in participants remained largely unchanged despite adherence to supplementation, suggesting limited iron absorption. This outcome implies that future studies would be useful to explore the relationship between iron supplementation and genital health, ideally in contexts where iron absorption is more effectively achieved 105.

### Coinfection with *Neisseria gonorrhoeae* or *Mycoplasma genitalium*

*C. trachomatis *infection often coincides with co-infections involving other bacterial STIs or bacterial vaginosis. Like *C. trachomatis*, symptomatic *Neisseria gonorrhoeae* infections are associated with CST IV. 106. The dynamics between *C. trachomatis *and *N. gonorrhoeae*, especially regarding nutrient availability and immune responses, are complex. In an *in vitro* study, Onorini *et al*. observed that *N. gonorrhoeae *co-infection reduced the size and quantity of *C. trachomatis* serovar E inclusions in HeLa cells and decreased chlamydial progeny yield 107. Ball *et al*. similarly found that *N. gonorrhoeae*-*C. trachomatis* serovar D co-infection hindered chlamydial EB recovery but not overall replication, with inclusions resembling aberrant bodies 103. Interestingly, tryptophan or indole supplementation with Fe(NO₃)₃ did not restore normal chlamydial development, indicating *N. gonorrhoeae *may promote chlamydial persistence through pathways independent of iron or tryptophan availability 108. Furthermore, these findings were not replicated when *Chlamydia muridarum*, the mouse-adapted strain of chlamydia, or *C. trachomatis *serovar L2 were studied, which suggested that the persistence phenotype in response to coinfection might be strain-specific 108. *C. muridarum* is a natural respiratory pathogen for mice, which when inoculated into the female mouse vagina, leads to ascending genital tract infection and disease that is very similar to humans.

Beyond nutrient competition, differences in host immune modulation may benefit *N. gonorrhoeae*. For instance, co-incubation of *C. trachomatis *serovar L2 with neutrophils enhances *N. gonorrhoeae* growth 104. This is potentially because *C. trachomatis *secretes Chlamydial Protease Activity Factor (CPAF), which paralyzes neutrophils and reduces their oxidative responses, potentially shielding *N. gonorrhoeae* in co-infected environments 109. While *N. gonorrhoeae* may benefit from* C. trachomatis’ *suppression of neutrophil function, *C. trachomatis *may leverage *N. gonorrhoeae*’s ability to impair adaptive immunity through promoting death of antigen-presenting cells and limiting T-cell proliferation 110.

Few *in vivo* studies exist on *C. trachomatis*-*N. gonorrhoeae* co-infections. A *C. muridarum*/*N. gonorrhoeae* co-infection mouse model showed an increased vaginal *N. gonorrhoeae* burden but no additional upper genital tract involvement 111. Co-infected mice exhibited higher levels of *N. gonorrhoeae* despite elevated neutrophil numbers, possibly because chlamydial infection reduced the expression of antimicrobial peptides, including cathelicidin-related antimicrobial peptide (CRAMP) and secretory leukocyte peptidase inhibitor (SLPI), that are effective against Gram-negative bacteria like *N. gonorrhoeae* 112. This suggests a potential role for these mediators in *N. gonorrhoeae* defense, which could be compromised by preexisting *C. trachomatis *infection 111.

Onorini *et al*. recently reported that *N. gonorrhoeae* enhancement in co-infection models requires active *C. muridarum* replication in the genital tract; mice that had cleared *C. muridarum* from the vagina but retained the bacteria in gut tissue showed no increase in vaginal *N. gonorrhoeae* abundance 113. Given that both *C. trachomatis *and *N. gonorrhoeae *express potent pro-inflammatory TLR2 ligands 39, neutrophil recruitment is likely proportional to the pathogen load at the infection site, with chlamydial CPAF synthesis contributing *in situ* to inhibition of neutrophil antimicrobial activity. However, a possible confounding factor in these studies is the difference between murine and human vaginal microbiomes 114,115,116,117 which limits the ability to identify human-relevant CVM members that might affect STI pathogenesis. Human studies are essential to better understand the metabolic dynamics in coinfections involving *C. trachomatis *and *N. gonorrhoeae*.

Due to interspecies differences, murine models may not effectively identify CVM factors relevant to human infections and their spread. For example, bacterial vaginosis is strongly linked with CST IV microbiomes 12,15 and increases the risk for bacterial STI infections in the cervix 118,119. However, the CVM composition’s role in upper genital tract infection remains underexplored. A study by Russell *et al*., which included data from 225 women at risk for STI infections in the T cell Response Against Chlamydia (TRAC) cohort, found that *N. gonorrhoeae *co-infection significantly heightened the risk of *C. trachomatis *ascending to the upper genital tract 120. *N. gonorrhoeae *¬ co-infection also increased the likelihood of new *C. trachomatis *infections over one year, possibly due to *N. gonorrhoeae*’s immunosuppressive effects, which compromise the generation of protective immune responses to *C. trachomatis *120. This agrees with *in vitro* data that reveal extracellular vesicles released from *N. gonorrhoeae *compromise dendritic cell activation 121, and *in vivo* data generated in the *N. gonorrhoeae *mouse model that show infection induces immune inhibitory TGF-ß production and regulatory T cells that compromise generation of Th1 responses 122, which are important for host defense against both bacterial pathogens. Finally, polymorphonuclear leukocytes (PMNs) are thought to facilitate *C. trachomatis *ascension 123,124, so *N. gonorrhoeae*’s promotion of PMN influx may indirectly contribute to *C. trachomatis *upward spread during co-infection. Together, these findings underline the complexity of *C. trachomatis*-*N. gonorrhoeae* interactions and underscore the need for human-specific studies to clarify how CVM composition and coinfection influence the pathogenesis of these STIs.

*Mycoplasma genitalium *is another STI pathogen frequently co-detected with *C. trachomatis *125. Unlike *C. trachomatis*, which is intracellular, *M. genitalium* exists extracellularly but shares a dependence on the host for biosynthesis of amino acids, nucleic acids, and fatty acids 126. A secondary analysis of the TRAC cohort indicated that women with *M. genitalium *infection in the cervix or endometrium had an elevated risk of histologic endometritis, particularly among those with endometrial *M. genitalium* infection 127. Additionally, detection of *C. trachomatis *in the endometrium was associated with a higher likelihood of detecting *M. genitalium* 127. A study by Dirks *et al*. using 1,673 urogenital samples, mostly from women (n = 1,121, 67%), found no correlation between *C. trachomatis *and *M. genitalium* cervical load in coinfected individuals 128. However, these findings have not been explored within the context of the CVM, which could influence the interaction between these pathogens.

Inconsistent findings from *in vitro*, murine, and human clinical studies on coinfections may be partially due to the absence of *in vivo* CVM studies, differences in the CVM between humans and mice, and the critical role of the immune response in mediating susceptibility to coinfection. Further human studies that investigate iron availability, other metabolites, or bacterial profiles associated with "high-risk" or "protective" CVMs—as well as comparisons of immune responses in individuals with single versus co-infection—are essential to better understand how pathogen coinfection influences infection dynamics and disease outcomes among these "partners in crime" 39.

### CVM compositions associated with *C. trachomatis* ascension

The composition of the CVM may also influence the risk of chlamydial infection spread, upper genital tract inflammation, and related immune pathology. *C. trachomatis *ascension to the uterus and Fallopian tubes is a prerequisite for development of PID 39. Cervical chlamydial load has been positively associated with an increased likelihood of infection ascension, as shown by both mathematical modeling 124 and the TRAC study which linked higher cervical *C. trachomatis *loads with increased risk of endometrial infection 120. Studies also correlate cervical *C. trachomatis *burden with an elevated risk of PID 129. CVM community members, particularly nutrient- or metabolite-producing microbes, may indirectly support chlamydial replication in the endocervical epithelium, thus increasing the likelihood of ascension. Additionally, the CVM may influence *C. trachomatis *ascension by modulating local inflammatory responses. For example, a diminished or delayed neutrophil influx could reduce immune-mediated clearance, allowing for prolonged infection. However, neutrophils have limited effectiveness in clearing *C. trachomatis *109 and may even facilitate its spread 124,130,131.

In the cervix, *C. trachomatis *triggers production of inflammatory chemokines and cytokines involved in recruiting immune cells, including T cells and macrophages, to the site of infection 132,133,134. While this response is critical for bacterial clearance, excessive or prolonged inflammation can damage epithelial integrity, potentially enhancing pathogen dissemination to the upper genital tract. Our lab recently observed a positive correlation between CVM-derived amplicon sequence variants and levels of seven cytokines previously associated with *C. trachomatis* ascension 129, suggesting that local immune responses during infection are not solely driven by *C. trachomatis* abundance. These cytokines—CXCL10, TNF-α, IL-17A, CXCL9, CXCL11, CCL4, and CXCL13 100—play roles in immune cell recruitment and barrier function. Their elevated levels in the presence of certain CVM compositions suggest that the microbiome may shape the host’s inflammatory environment in ways that impact *C. trachomatis* pathogenesis.

In recent work analyzing TRAC cohort data via 16S rRNA sequencing, our lab examined if CVM composition predicts *C. trachomatis *ascension. Previously, *N. gonorrhoeae *co-infection, and high *C. trachomatis *burden has been associated with increased ascension 120. For some CSTs, we observed that it was possible to discriminate between participants with or without ascending *C. trachomatis *infection 103. CST IV correlated with ascension, highlighting an interaction between CVM composition and *C. trachomatis *infection severity. Although many CST IV microbes are indole producers, which generally aids *C. trachomatis *survival, some *Prevotella* species dominant in CST IV do not produce indole. This underscores the need to look beyond species identification and focus on metabolite production when analyzing CVM data. Our study also noted lower *Sutterella* *spp. *levels in endometrial-positive women. Since *Sutterella* *spp. *have been associated with heightened Th17 responses 135,136, the reduction of *Sutterella* *spp*. in endometrial-positive *C. trachomatis *cases might indicate decreased Th17 activity, impacting this potential mechanism of chlamydial host defense 137.

### CVM compositions associated with duration of *C. trachomatis* infection

Natural, or “spontaneous,” clearance of *C. trachomatis *infection occurs in an estimated 11%-44% of cases 138. This phenomenon is defined as the resolution of infection between an initial *C. trachomatis*-positive test and a follow-up visit prior to antibiotic treatment, which can range from days to several weeks 139. Spontaneous clearance has been associated with lower tryptophan levels in the CVM, though it is unclear if this results from reduced indole-producing microbes or reflects elevated IFNγ levels associated with an effective adaptive immune response 138. Research also suggests that a *Lactobacillus*-dominated CVM may support spontaneous clearance; women with a predominance of *Lactobacillus spp.* were more likely to test negative for *C. trachomatis *at a follow-up appointment 140. Furthermore, women who naturally cleared *C. trachomatis *tended to have lower initial *C. trachomatis *burdens, and *L. crispatus* abundance was inversely correlated with *C. trachomatis *load, reinforcing the role of CVM composition in chlamydial clearance 76.

However, *C. trachomatis *infection can become chronic, lasting for a year or longer in up to 40% of women 141, which increases the risk of long-term sequelae. Chronic infection may be sustained by intermittent periods of non-replicating, aberrant *C. trachomatis *forms that reinitiate active replication when immune pressure decreases or when the CVM provides a nutrient-rich environment. As a result, CVM characteristics—such as high levels of indole-producing microbes—could indicate an elevated risk of chronic infection. Similarly, recurrent *C. trachomatis *infection can pose a challenge for some patients. A recent study of cohort 560 Black and Hispanic women found that those with both bacterial vaginosis and CST IV had an increased risk of *C. trachomatis* infection, and a higher likelihood of reinfection after treatment 142. In this CVM subgroup, which was linked to both acquisition and reinfection, the predominant species included *Candidatus Lachnocurva vaginae*, *Prevotella*, and *Acinetobacter* 142. Notably, *Candidatus Lachnocurva vaginae* showed the strongest association with *C. trachomatis*, but it was also highly correlated with ~nine other species, including *Prevotella *and *Acinetobacter*. The strong co-occurrence of these *C. trachomatis*-associated species within the same CVM suggests they CVM functions as a polymicrobial network that enhances the risk of both infection and reinfection. This underscores the need for future studies to consider polymicrobial interactions in chlamydial pathogenesis.

## IMPLICATIONS FOR CERVICOVAGINAL MICROBIOME RESEARCH

### Study cohorts and techniques 

Research on the CVM is rapidly advancing, and the field is constantly adapting and improving its methodologies. Sequencing techniques vary across studies, each presenting unique limitations and considerations. Currently, 16S rRNA amplicon sequencing is the most widely used approach in CVM composition research. Advances in 16S rRNA sequencing have enhanced the resolution and accuracy of microbiome identification, as reviewed recently 143. Qing *et al*. evaluated the range of methods employed in CVM research, specifically comparing different 16S rRNA sequencing techniques. Their study concluded that a sequencing pipeline targeting the V1-V3 variable region of the genome currently offers the most optimal and accurate taxonomic resolution of microbial communities relevant to CVM research 144. It is unrealistic to expect that CVM-derived 16S rRNA libraries will always reflect the presence of STI pathogen(s), so highly sensitive, clinical diagnostics should be used in determining infection status of study participants. Misclassification with respect to infection status and/or outcome 145 has potential to confound bioinformatic analyses.

Early CVM studies used less optimal sequencing techniques, and even the best 16S rRNA sequencing methods can offer only taxonomic information, often struggling to differentiate closely related species. Metagenomic approaches, which can identify microbial strains at the species level, provide additional specificity and improve accuracy in identifying CVM community members 146. Multiomic techniques such as transcriptomics 147, proteomics 148, and metabolomics 77,149 are also emerging, offering functional insights and more comprehensive information on population dynamics. For instance, Borgogna *et al*.’s investigation of the metabolomic profiles of 145 women, with and without *C. trachomatis *or *M. genitalium* coinfection, identified a distinct metabolome in infected women, marked by elevated LCFAs compared to healthy individuals 77. Additionally, many CVM bacterial species are fastidious or uncultivable, posing challenges for *in vitro* and *in vivo* studies aiming to create complex microbial communities that accurately mimic the human CVM. To address this, novel methods like culturomics have been developed to more accurately model this complex ecosystem 150. Culturomics is a technique that employs a diverse range of bacterial growth media and conditions to cultivate microorganisms that are typically difficult to grow under standard bacterial growth conditions 151,152. Furthermore, not all CVM members can be detected by 16S rRNA analysis, including bacteriophages and viruses. Despite their lack of representation in 16s rRNA microbiome studies, these microorganisms can significantly impact the microbiome. For example, bacteriophages have the potential to modulate bacterial communities while eukaryotic viruses can alter local host immune responses which, in turn, may influence CVM composition 153,154,155.

Designing clinical studies on the CVM requires careful consideration of cohort characteristics. Unlike the more stable gut microbiome, the CVM is highly dynamic, influenced by factors such as hormonal fluctuations, menstruation, sexual activity, hygiene, and contraceptive use 156. Due to these influences, longitudinal cohort studies are essential to capture CVM changes over time, rather than relying on one-time snapshots. This approach is particularly important for studying the CVM's role in STIs, as it allows comparisons of CVM composition before and after infection onset. Monitoring pre-existing immunity within cohorts can further reveal how immune responses interact with the CVM in the context of *C. trachomatis *acquisition and progression. However, longitudinal studies face challenges like participant retention, recruitment bias, and cost 157, though anticipated clinical trials for vaccines could provide valuable cohorts for such investigations.

Sample collection and preparation also impact study outcomes. Differences in storage solutions (e.g., GeneLock, RNAlater, SPG media) and sampling methods (e.g., self-swabs, vaginal sponges) can affect downstream analyses 158,159,160. Some collection methods may introduce contaminants—such as propylene glycol from lubricants, which can interfere with mass spectrometry 161,162. Additionally, certain vaginal lubricants contain antimicrobials, which may skew microbiome results. For immune studies, cytometry by time-of-flight (CyTOF) is useful but highly susceptible to barium contamination. This is particularly relevant as recent research has shown that tampons can introduce trace metals, including barium, into samples, potentially impacting CVM studies 163. As CVM research advances, the development of standardized techniques and optimized models will be crucial for improving consistency and accuracy.

### Clinical applications

A major goal of CVM research is to apply knowledge about the host-microbiome-pathogen relationship to reproductive health treatments. Inspired by the success of fecal microbiota transplants, vaginal microbiota transplants (VMTs) are being investigated 156. For instance, VMTs have shown promise in treating intractable bacterial vaginosis; a small study reported remission in 4 of 5 patients following VMT 164, and larger studies are ongoing 165. This approach may also hold potential for STI treatments, including *C. trachomatis*, by restoring protective *Lactobacillus* species in the CVM to promote eubiosis and potentially mitigate disease. However, achieving durable changes in the CVM microbiome is challenging, because even beneficial *Lactobacillus* species face competitive pressures from other resident bacteria 71, which complicates the development of long-term treatments.

## CONCLUSION

We summarized studies examining how the CVM and co-pathogens affect the risk of *C. trachomatis *acquisition and infection spread in women (**Figure 1**). It is evident that the intricate interactions between the CVM, host immune responses, and pathogenic microorganisms - particularly *C. trachomatis *- drive clinical outcomes. These factors underscore the critical role of microbiome composition in reproductive health and disease. The CVM influences *C. trachomatis *pathogenesis both directly and indirectly. The contrasting influences of metabolites such as SCFAs and LCFAs in the gut and CVM, highlight the complexity of microbial and host dynamics. Additionally, the potential adaptations of *C. trachomatis *to nutrient and immune-mediated pressures reveal novel mechanisms that enable its survival and pathogenesis.

Emerging research methodologies, including multiomic analyses, are paving the way for deeper insights into these complex relationships. For example, a new multi-cell type microfluidic chamber called "vagina-on-a-chip" allows for controlled *in vitro* studies of CVM microbial effects on vaginal epithelial cells 166. Likewise, an *in vivo* mouse model that better replicates the human CVM would be invaluable for exploring CVM-pathogen-host interactions 167. Adding metabolomics data in studies could also improve our understanding of these complex relationships 33,77,149. However, challenges such as methodological variability, cohort differences, and limitations in current model systems underscore the need for standardized techniques and translational studies. Furthermore, the development of innovative clinical applications, such as vaginal microbiota transplants, offers promising avenues for improving outcomes in microbial dysbiosis and STIs.

Ultimately, advancing our understanding of the CVM's role in health and disease will require collaborative efforts that integrate microbiology, immunology, and reproductive health. Such efforts hold the potential to serve as a biomarker source for assessing STI risk and disease progression, and to drive development of more effective prevention and therapeutic strategies, improving health outcomes for individuals globally.

Our microbiome-focused studies are supported by the National Institute of Allergy and Infectious Diseases via U19 AI084024 (Darville, PI) and R01AI170959 (O’Connell, PI). Emily Hand is funded in part by NIGMS T32 grant 5T32GM149370. Indriati Hood-Pishchany is funded in part through grants from the Bill and Melinda Gates Foundation (Awards No. INV-055566 and INV-072197).

## CONFLICT OF INTEREST

The authors have declared that no conflicts of interest exist.
